# Rapid Exercise-Induced Mobilization of Dendritic Cells Is Potentially Mediated by a Flt3L- and MMP-9-Dependent Process in Multiple Sclerosis

**DOI:** 10.1155/2015/158956

**Published:** 2015-10-29

**Authors:** Nathalie Deckx, Inez Wens, Amber H. Nuyts, Wai-Ping Lee, Niel Hens, Gudrun Koppen, Herman Goossens, Pierre Van Damme, Zwi N. Berneman, Bert O. Eijnde, Nathalie Cools

**Affiliations:** ^1^Laboratory of Experimental Hematology, Vaccine & Infectious Disease Institute (VAXINFECTIO), Antwerp University Hospital, Faculty of Medicine and Health Sciences, University of Antwerp, 2610 Wilrijk, Belgium; ^2^REVAL Rehabilitation Research Centre, BIOMED Biomedical Research Institute, Faculty of Medicine and Life Sciences, Hasselt University, 3590 Diepenbeek, Belgium; ^3^Interuniversity Institute for Biostatistics and Statistical Bioinformatics (I-BIOSTAT), Hasselt University, 3590 Diepenbeek, Belgium; ^4^Centre for Health Economic Research and Modelling Infectious Diseases (CHERMID), Vaccine & Infectious Disease Institute (VAXINFECTIO), Faculty of Medicine and Health Sciences, University of Antwerp, 2610 Wilrijk, Belgium; ^5^Flemish Institute of Technological Research (VITO), Unit Environmental Risk and Health, 2400 Mol, Belgium; ^6^Laboratory of Medical Microbiology, Vaccine & Infectious Disease Institute (VAXINFECTIO), Faculty of Medicine and Health Sciences, University of Antwerp, 2610 Wilrijk, Belgium; ^7^Centre for the Evaluation of Vaccination, Vaccine & Infectious Disease Institute (VAXINFECTIO), Faculty of Medicine and Health Sciences, University of Antwerp, 2610 Wilrijk, Belgium

## Abstract

In healthy individuals, one exercise bout induces a substantial increase in the number of circulating leukocytes, while their function is transiently suppressed. The effect of one exercise bout in multiple sclerosis (MS) is less studied. Since recent evidence suggests a role of dendritic cells (DC) in the pathogenesis of MS, we investigated the effect of one combined endurance/resistance exercise bout on the number and function of DC in MS patients and healthy controls. Our results show a rapid increase in the number of DC in response to physical exercise in both MS patients and controls. Further investigation revealed that in particular DC expressing the migratory molecules CCR5 and CD62L were increased upon acute physical activity. This may be mediated by Flt3L- and MMP-9-dependent mobilization of DC, as demonstrated by increased circulating levels of Flt3L and MMP-9 following one exercise bout. Circulating DC display reduced TLR responsiveness after acute exercise, as evidenced by a less pronounced upregulation of activation markers, HLA-DR and CD86, on plasmacytoid DC and conventional DC, respectively. Our results indicate mobilization of DC, which may be less prone to drive inflammatory processes, following exercise. This may present a negative feedback mechanism for exercise-induced tissue damage and inflammation.

## 1. Introduction

In general, physical activity or exercise improves health and reduces the risk of developing several diseases, like cardiovascular disease and type II diabetes. These health-promoting effects result from the immediate and long-term influence of exercise on many of the human body's systems, including the cardiovascular system, the energy systems, and the immune system. In a healthy population, one exercise bout induces a substantial increase in the number of circulating leukocytes. These effects are related to both the duration and intensity of the physical activity, with high-intensity exercise yielding the most profound effects [1, 2]. Paradoxically, one bout of physical activity has a temporary suppressive effect on the function of both innate and adaptive immune cells, for example, neutrophils [3], monocytes [4, 5], macrophages [6], natural killer cells [7], T-cells [8], and B-cells [9].

The effect of acute exercise on patients with immune-mediated health conditions, such as multiple sclerosis (MS), is much less studied. MS is a chronic demyelinating, inflammatory disease of the central nervous system (CNS) [10], predominantly affecting young adults in their most productive years. Based on previous efforts focusing on the role of the adaptive immune system in the pathogenesis of MS, it is currently well established that autoreactive T helper type 1 (Th1) and Th17 cells mediate the inflammatory processes in the CNS [11, 12]. Recent evidence also suggests involvement of innate immunity, including dendritic cells (DC), in the initiation and maintenance as well as progression of MS [13, 14].

In human blood, two major subsets of DC have been identified, namely myeloid or conventional DC (cDC) and plasmacytoid DC (pDC) [15]. They are characterized by a difference in the expression profile of cytokine receptors and cytokines [16–19], of migratory markers and migration potential [20] and of Toll-like receptors (TLR) [21]. Since DC have the unique capacity to polarize the differentiation of T-cells, they are central in regulating the balance between inflammation and tolerance [22]. For this, DC continuously capture antigens from the environment, process them, and present them on the cell-surface complexed to major histocompatibility (MHC) molecules, for example, human leukocyte antigen- (HLA-) DR. Together with context-dependent expression of costimulatory molecules, such as CD86, and secretion of cytokines, DC can induce either effector T-cells or regulatory T-cells (Treg).

Previously, we demonstrated that significantly lower percentages of circulating DC are found in the peripheral blood of MS patients carrying MS-associated genetic risk factors [23]. These reduced levels of DC may reflect enhanced trafficking from the blood towards the CNS. Indeed, whereas the capacity of DC to migrate to the sites of inflammation is regulated by the expression of chemokines and chemokine receptors, we have reported increased proportions of circulating cDC and pDC positive for the migratory molecule C-C chemokine receptor 5 (CCR5) in MS patients. Although this suggests that DC may drive the inflammatory response in MS, additional processes are likely to be involved, as indicated by the fact that circulating DC subsets were still significantly lower when comparing MS patients and healthy controls both carrying the same MS-associated genetic risk factor [23].

The objective of current study was to investigate whether one exercise bout could increase and mobilize DC rapidly in the peripheral blood in healthy controls and MS patients. Moreover, the function of DC following acute physical activity was evaluated in both healthy controls and MS patients.

## 2. Material and Methods

### 2.1. Study Population

A total of 22 MS patients, diagnosed according to the revised McDonald criteria [24] and aged > 18 years, were recruited. In addition, 9 control subjects that were matched for gender, age, and body mass index (BMI) were included in the study (Table 1). Subjects were excluded if they had an expanded disability status scale (EDSS) score > 6, that is, not being able to walk 100 m without walking aid, a diagnosis of diabetes mellitus type II, other autoimmune diseases (diabetes mellitus type I and/or rheumatoid arthritis), and other chronic diseases (cardiovascular, pulmonary, and/or renal), were pregnant, participated in another study, had contraindications to perform physical activity, received corticosteroid treatment 3 months prior to the start of the study, or had an acute MS exacerbation 6 months prior to the start of the study. Patient and control characteristics and medication use are depicted in Supplementary Table 1 (in Supplementary Material available online at http://dx.doi.org/10.1155/2015/158956). All subjects gave informed consent in accordance with the Declaration of Helsinki and the protocol was approved by the local Ethics Committees of Hasselt University and of the Antwerp University Hospital.

### 2.2. Study Design

All patients and controls performed an acute physical exercise test using Technogym training equipment (LJ Capelle aan den IJssel, The Netherlands). The exercise bout started with a moderate-to-high-intensity endurance exercise, including 15 min of cycle ergometry followed by a 15 min walking session during which study subjects must have reached their individually calculated heart rate (HR) = [resting HR + 65%  *∗*  (maximum HR − resting HR)]. The resting HR was measured horizontally after 15 min of rest. The maximum HR was calculated by subtracting their age from 220. Next, the study subjects performed a moderate-to-high-intensity resistance exercise, including unilateral leg strength training (leg press, leg extension, and leg curl) and bilateral arm strength training (chest press, latissimus pull, arm curl) consisting of 3 × 10 repetition sets that were interspersed by 2 min rest intervals, during which study subjects trained at 70% of 1 repetition maximum (RM). Venous blood was collected before the exercise bout, immediately after the bout and 2 hours after the bout in both heparin and serum-separating tubes (BD Biosciences, Erembodegem, Belgium).

### 2.3. Isolation and Stimulation of Leukocytes

Leukocyte cell counts were measured using an automated cell counter (ABX Micros 60, Horiba, Deurne, Belgium). Next, leukocytes were isolated using density gradient purification (Ficoll Paque PLUS, GE Healthcare, Chalfont St Giles, UK) for* ex vivo* flow cytometric analysis of DC and Treg subsets. For evaluation of Treg numbers using intracellular cytokine staining, leukocytes were additionally treated with 10 *μ*g/mL brefeldin A (Life Technologies, Merelbeke, Belgium) for 16–18 hours. Subsequently, leukocytes were fixed and permeabilized using a fixation and permeabilization concentrate and diluent, according to the manufacturer's instructions (eBioscience, Vienna, Austria). Simultaneously, 1 mL of peripheral blood was (i) stimulated overnight with 2 *μ*g/mL lipopolysaccharide (LPS), a TLR4 ligand (Invivogen, Toulouse, France), and 50 *μ*g/mL interferon-*γ* (IFN-*γ*) (Immunotools, Friesoythe, Germany) or (ii) stimulated overnight with 10 *μ*g/mL imiquimod (IQ), a TLR7 ligand (Invivogen), or (iii) left untreated as a control. Plasma was collected and stored at −20°C for batch analysis of TLR-mediated cytokine production. Next, leukocytes were enriched after red blood cell lysis (0.155 M NH_4_Cl, 0.01 M KHCO_3_, and 0.1 mM Na_2_-EDTA) for evaluation of DC activation state and chemokine responsiveness.

### 2.4. Flow Cytometry

Immunophenotyping of DC was done by direct immunofluorescence staining using the following fluorochrome-labeled mouse anti-human monoclonal antibodies: anti-blood dendritic cell antigen-1 (BDCA-1) phycoerythrin (PE) (Miltenyi Biotec, Leiden, The Netherlands), anti-BDCA-2 allophycocyanin (APC) (Miltenyi Biotec), anti-lineage I (Lin I; anti-CD3, anti-CD14, anti-CD16, anti-CD19, anti-CD20, and anti-CD56) fluorescein isothiocyanate (FITC) (BD Biosciences), anti-CD62L PE-cyanine 7 (PE-Cy7) (eBioscience), anti-CD86 V450 (BD Biosciences), anti-CCR5 PE-Cy7 (BD Biosciences), and anti-HLA-DR APC-H7 (BD Biosciences) antibodies.

Treg subsets were characterized using the following fluorochrome-labeled mouse anti-human monoclonal antibodies: anti-CD3 peridinin chlorophyll protein Cy5.5 (PerCP Cy5.5) (BD Biosciences), anti-CD4 APC-H7 (BD Biosciences), anti-CD8 pacific blue (PB) (Life Technologies), anti-CD25 PE-Cy7 (BD Biosciences), anti-IL-10 APC (BD Biosciences), anti-transforming growth factor-*β* (TGF-*β*) (IQ Products, Groningen, The Netherlands), and anti-forkhead box P3 (FoxP3) Alexa 488 (BD Biosciences).

In all flow cytometric assays, dead cells were excluded by addition of violet live/dead stain (Life Technologies) to the antibody mixture. Fluorescence minus one in combination with nonreactive isotype-matched antibodies was used as control. For analytical flow cytometry, at least 10^5^ events were measured using a CyFlow ML flow cytometer (Partec, Münster, Germany). All results were analyzed using FlowJo software (Tree Star, Inc., Ashland, OR, USA).

### 2.5. Soluble Analyte Secretion Assays

Serum was isolated using serum-separating tubes (BD Biosciences). Serum levels of matrix metalloproteinase-9 (MMP-9) (Meso Scale Discovery, Rockville, MD, USA), macrophage inflammatory protein-1*α* (MIP-1*α*) (eBioscience), and Fms-related tyrosine kinase 3 ligand (Flt3L, R&D, Minneapolis, MN, USA) were quantified using ELISA according to manufacturer's instructions.

For quantitative detection of the cytokines secreted following stimulation of peripheral blood, collected plasma samples were analyzed using the following commercially available ELISA kits: IL-1*β*, IL-6, IL-12p70, TNF-*α*, IFN-*α* (PBL InterferonSource, Piscataway, NJ, USA), MMP-9, and caspase-1 (R&D), according to manufacturer's instructions. All kits were purchased from eBioscience, unless stated otherwise.

### 2.6. Statistical Analysis

All data were analyzed using SAS 9.3 software (SAS Institute Inc., Cary, USA). Linear mixed models were used to analyze repeated measures data [25]. A model was built stepwise per outcome variable, starting from a univariate model with “time” as the only fixed effect. New models were constructed, step by step, by adding other fixed effect variables including MS type, gender, age, BMI, EDSS, and MS-specific medication. The variable “MS type” included relapsing-remitting (RR) and chronically progressive (CP) MS patients. The variable “MS-specific medication” included untreated patients, 1st-line treatment, and 2nd-line treatment. Since the variables “EDSS,” “MS type,” and “MS-specific medication” have no value in healthy individuals, we added an indicator variable to discriminate between MS patients and healthy controls. A *P* value < 0.10 (*F* test) was used as the threshold for retaining a fixed effect during model building to decrease the chance of missing a significant effect in the final model. Subsequently, possible interaction effects between the retained fixed effects were assessed and also retained if *P* < 0.10. For interpretation of the fixed effects in the final model, the threshold for statistical significance was set at *P* < 0.05. Since we aim to investigate temporal effects, we only report main and interaction effects of time, that is, the effect of one exercise bout. Models consisting of more than 12 parameters were not interpreted because of the risk of overfitting. When of interest,* post hoc* analyses were performed using contrast and subgroup analyses, that is, applying the Scheffé and the Bonferroni correction, respectively. Diagnostics were based on the studentized residuals and response variables were logarithmically transformed when necessary. Graphs were generated in GraphPad version 5 software (Prism, La Jolla, CA, USA). All data are presented as mean ± standard error of the mean (SEM).

## 3. Results

### 3.1. One Exercise Bout Induces a Profound Increase in the Number of Circulating Leukocytes

**Figure 1:**
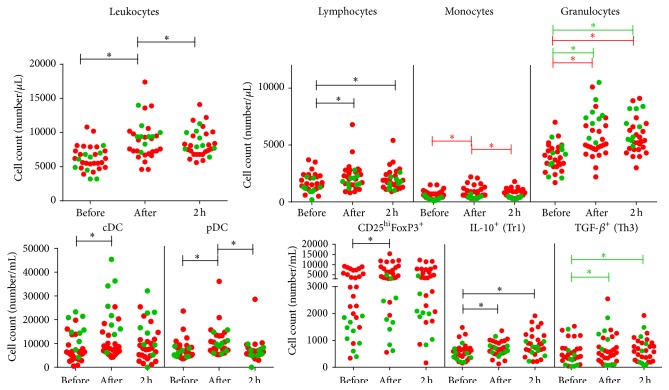
Leukocyte counts are increased upon physical exercise. Enumeration of the absolute number of immune cells was done by means of a double platform method using an automated cell counter and flow cytometry in healthy controls (green dots) and MS patients (red dots). Results are shown as mean absolute number ± SEM. Significance (^*∗*^ *P* < 0.05) in the complete population of both patients and controls is annotated by black lines, while green and red lines annotate significance in controls and patients, respectively. Abbreviations used are as follows: MS: multiple sclerosis; cDC: conventional dendritic cells; pDC: plasmacytoid dendritic cells; Treg: regulatory T-cell; FoxP3: forkhead box P3; IL: interleukin; Tr1: type 1 regulatory T-cell; TGF: transforming growth factor; Th3: T helper type 3 cell; before: measurement before the exercise test; after: measurement immediately after the test; 2 h: measurement 2 hours after the test; and SEM: standard error of the mean.

Since it was previously demonstrated by others that acute physical activity alters the number and function of circulating cells of the immune system [1], we first investigated the absolute number of circulating leukocytes and leukocyte subsets following one exercise bout in both MS patients (*n* = 22) and healthy controls (*n* = 9). Both study groups showed a rapid and immediate increase in absolute leukocyte numbers upon one exercise bout (*P* < 0.001) that normalized after two hours of recovery (*P* = 0.020, Figure 1). An immediate increase in the absolute number of lymphocytes (*P* < 0.001) was found upon one exercise bout, which did not recover after a 2-hour resting phase (*P* = 0.020, Figure 1). A distinct response between patients and controls was found for the absolute number of monocytes (*P* < 0.001) and granulocytes (*P* < 0.001) following the exercise bout (Figure 1). More specifically, whereas patients display an immediate increase in the monocyte count (*P* < 0.001), which recovers after the 2-hour resting phase (*P* < 0.001), no response to one exercise bout was observed in healthy controls. Patients and controls showed an immediate increase in the absolute granulocyte number after one exercise bout (MS: *P* < 0.001. Controls: *P* < 0.001), although this response was significantly higher in healthy controls as compared to MS patients. Furthermore, granulocyte numbers did not recover after the 2-hour recovery period in any of the study groups (MS: *P* < 0.001. Controls: *P* < 0.001). Noteworthy, the higher the patient's EDSS score, the less pronounced the increase in leukocytes (*P* < 0.001) and monocyte numbers (*P* < 0.001) following one acute exercise (Supplementary Figure 1).

Next, we investigated the absolute number of circulating DC subsets, namely cDC (Lin^−^ BDCA-1^+^) and pDC (Lin^−^ BDCA-2^+^). In both patients and controls, the cDC and pDC count increased rapidly and immediately after one exercise bout (cDC: *P* < 0.001, pDC: *P* < 0.001, Figure 1). Following a 2-hour recovery period, pDC counts normalized to steady-state levels (*P* < 0.001), whereas no significant drop to steady-state levels could be demonstrated for the cDC number.

Also the number of naturally occurring CD25^hi^FoxP3^+^ Treg (*P* < 0.001) and antigen-induced IL-10-producing type 1 Treg (Tr1) (*P* = 0.031) increased immediately after one exercise bout in both patients and controls (Figure 1). The number of Tr1 remained increased up to 2 hours after the exercise bout (*P* < 0.001). In addition, a significantly different response for the number of TGF-*β*-producing T helper 3 (Th3) cells was observed between patients and controls (*P* = 0.037, Figure 1). Whereas an immediate increase in the number of Th3 cells following one exercise bout was observed in healthy controls (*P* = 0.006), which remained high up to two hours after exercise (*P* = 0.010), no effect of the exercise bout on the Th3 cell count in patients could be demonstrated.

### 3.2. One Exercise Bout Induces Migration-Dependent Accumulation of DC in the Peripheral Blood

**Figure 2:**
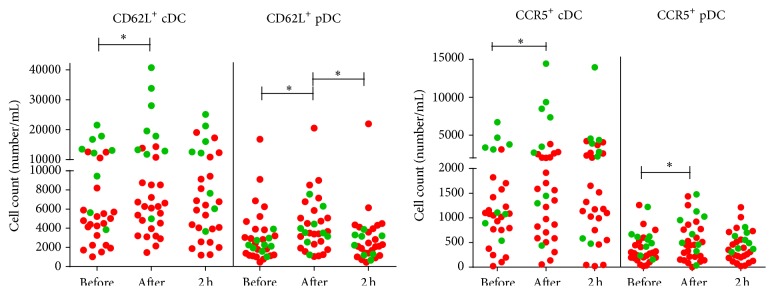
Increased numbers of cDC and pDC expressing the migratory molecules, CD62L and CCR5, upon one exercise bout. The migratory profile of DC was assessed by enumerating cDC and pDC expressing CD62L and CCR5 in healthy controls (green dots) and MS patients (red dots). Results are shown as mean absolute number ± SEM. Significance (^*∗*^ *P* < 0.05) in the complete population of both patients and controls is annotated by black lines. Abbreviations used are as follows: MS: multiple sclerosis; cDC: conventional dendritic cells; pDC: plasmacytoid dendritic cells; CD62L: CD62 ligand; CCR: C-C chemokine receptor; before: measurement before the exercise test; after: measurement immediately after the test; 2 h: measurement 2 hours after the test; and SEM: standard error of the mean.

Previous studies demonstrated that migration of leukocyte subsets, including DC, from and towards the peripheral blood is regulated by distinct signals, chemokines, and chemokine receptors [26]. Here, we observed that the absolute number of cDC and pDC expressing the cell adhesion molecule CD62 ligand (CD62L) increased immediately after one exercise bout in patients and controls (cDC: *P* = 0.017; pDC: *P* = 0.002, Figure 2). After two hours of rest, the number of CD62L^+^ cDC did not significantly recover to steady-state values, while the number of CD62L^+^ pDC did (*P* < 0.001). Furthermore, the absolute number of cDC and pDC expressing CCR5 increased immediately after one exercise bout in patients and controls (cDC: *P* = 0.024; pDC: *P* < 0.001; Figure 2) but failed to significantly return to steady-state values after two hours of recovery.

**Figure 3:**
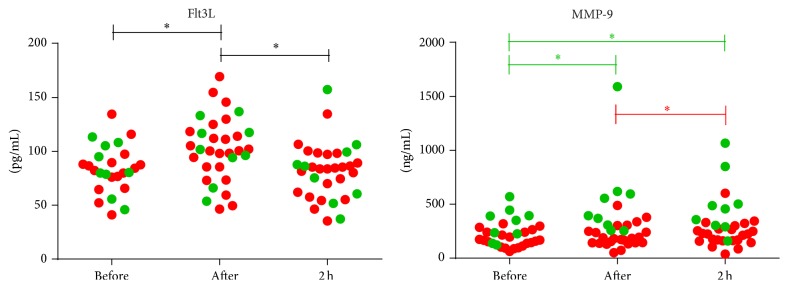
Induction of Flt3L and MMP-9 upon physical exercise. The serum level of Flt3L and MMP-9 was measured by means of ELISA in healthy controls (green dots) and MS patients (red dots). Results are shown as mean concentration ± SEM. Significance (^*∗*^ *P* < 0.05) in the complete population of both patients and controls is annotated by black lines, while green and red lines annotate significance in controls and patients, respectively. Abbreviations used are as follows: MS: multiple sclerosis; Flt3L: Fms-related tyrosine kinase 3 ligand; MMP: matrix metalloproteinase; before: measurement before the exercise test; after: measurement immediately after the test; 2 h: measurement 2 hours after the test; and SEM: standard error of the mean.

Given the pronounced effect of one exercise bout on DC expressing migratory markers together with the fact that MIP-1*α*, the ligand for CCR5, induces mobilization of DC precursors [27], we quantified the serum levels of signals involved in cell migration. In our hands, we were not able to detect any MIP-1*α* in the serum of patients and controls (data not shown). However, the serum level of Flt3L, a growth factor and a key regulator of DC homeostasis [28], immediately increased following one exercise bout in MS patients and healthy controls (*P* < 0.001, Figure 3). Following two hours of rest, Flt3L concentration significantly normalized to steady-state values (*P* < 0.001). In addition, patients and controls responded differently to one exercise bout with regard to the serum level of MMP-9 (*P* = 0.008, Figure 3), a cell migration-associated proteinase [29]. While the MMP-9 serum concentration immediately increased upon acute physical activity in controls (*P* = 0.009) and remained high up to two hours after the exercise bout (*P* = 0.026), patients only showed a slight, but significant, increase after the 2-hour recovery period (*P* = 0.028).

### 3.3. Reduced TLR Responsiveness of DC upon Acute Physical Exercise

**Figure 4:**
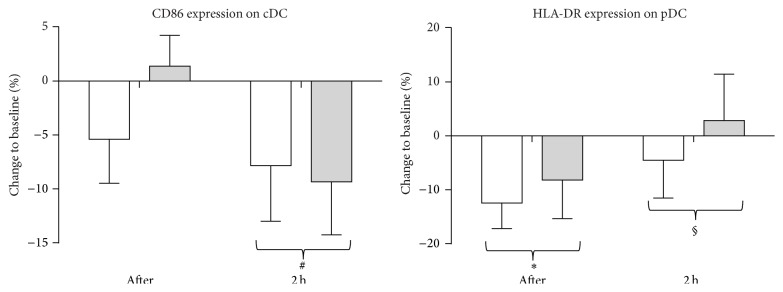
Decreased activation state of DC upon TLR stimulation after one exercise bout in healthy controls (white bars) and MS patients (grey bars). MFI of CD86 and HLA-DR expression on cDC and pDC after LPS and IFN-*γ* or IQ stimulation, respectively, was measured using flow cytometry. Mean fold changes are calculated as the ratio between the MFI in the stimulated condition and the nonstimulated condition. Then, % changes after exercise and after 2 hours of rest with respect to the baseline value are calculated. Results are shown as mean % change ± SEM. ^*∗*^ *P* < 0.05 for the difference between results before and after the test. # indicates a significant effect between before and 2 hours after the test. § indicates a significant effect between after and 2 hours after the test. Abbreviations used are as follows: cDC: conventional dendritic cells; pDC: plasmacytoid dendritic cells; LPS: lipopolysaccharide; IFN: interferon; IQ: imiquimod; HLA: human leukocyte antigen; MFI: mean fluorescence intensity; after: measurement immediately after the test; 2 h: measurement 2 hours after the test; and SEM: standard error of the mean.

In order to assess the effect of one exercise bout on the activation state and chemokine receptor responsiveness of circulating DC in an inflammatory microenvironment, blood samples were stimulated with a TLR4 ligand, LPS, in combination with IFN-*γ*, or a TLR7 ligand, IQ. Both ligands are known to activate cDC and pDC, respectively [30, 31]. Using flow cytometry, we measured the fold change in the expression level of CCR5, CD86, and HLA-DR on cDC and pDC upon TLR stimulation. In both patients and controls, CD86 expression is upregulated on cDC following stimulation with LPS and IFN-*γ*. However, the upregulation of this costimulatory marker was significantly less pronounced two hours after the exercise bout as compared to steady-state values (*P* = 0.009, Figure 4). On pDC, the upregulation of the expression of HLA-DR following IQ stimulation was significantly less pronounced immediately after the exercise bout in patients and controls (*P* = 0.011, Figure 4), whereas it normalized again after a 2-hour recovery period (*P* = 0.043). No effect of acute exercise following TLR stimulation could be demonstrated for the expression levels of CCR5 and HLA-DR on cDC and of CCR5 and CD86 on pDC.

**Figure 5:**
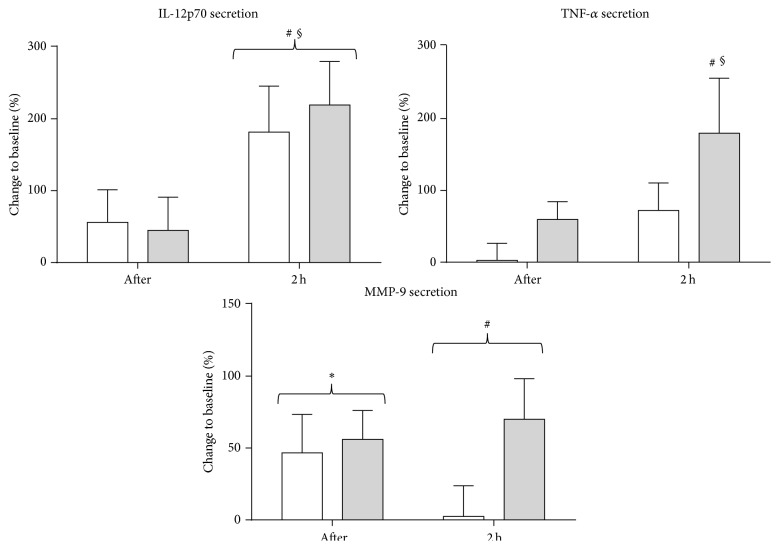
Increased secretion of inflammatory mediators upon TLR stimulation after one exercise bout. Blood samples of healthy controls (white bars) and MS patients (grey bars) were (i) stimulated overnight with LPS in combination with IFN-*γ* or (ii) left unstimulated. Secretion of inflammatory mediators was quantified using ELISA. For the secretion of TNF-alpha, only patients on 2nd-line treatment are depicted. Mean fold changes are calculated as the ratio of the secreted concentration in the stimulated condition to the nonstimulated condition. Then, % changes after exercise and after 2 hours of rest with respect to the baseline value are calculated. Results are shown as mean % change ± SEM. ^*∗*^ *P* < 0.05 for the difference between results before and after the test. # indicates a significant effect between before and 2 hours after the test. § indicates a significant effect between after and 2 hours after the test. Abbreviations used are as follows: LPS: lipopolysaccharide; IL: interleukin; TNF: tumour necrosis factor; IFN: interferon; MMP: matrix metalloproteinase; after: measurement immediately after the test; 2 h: measurement 2 hours after the test; and SEM: standard error of the mean.

Next, also the secretion of inflammatory mediators upon TLR stimulation was assessed. We observed significantly larger amounts of IL-12p70 released upon LPS and IFN-*γ* stimulation two hours after the exercise bout in patients and controls (Figure 5) as compared to steady-state secretion levels (*P* < 0.001) and to secretion levels immediately after the exercise bout (*P* < 0.001), whereas no effect on IL-12p70 secretion in patients and controls was found immediately after the exercise bout. No effect on TNF-*α* secretion upon LPS and IFN-*γ* stimulation could be demonstrated immediately after acute exercise in both patients and controls. TNF-*α* secretion upon LPS and IFN-*γ* stimulation was significantly influenced by the treatment regimen of the study groups (*P* = 0.005). Two hours after the exercise bout, a significant increase in the secretion of TNF-*α* was found following LPS and IFN-*γ* stimulation as compared to steady-state secretion levels (*P* < 0.001) and to secretion levels immediately after the exercise bout (*P* = 0.007) in patients with 2nd-line treatment, while this could not be demonstrated in untreated patients and patients with 1st-line treatment. Also in controls a trend towards an increase in TNF-*α* secretion upon LPS and IFN-*γ* stimulation two hours after the exercise bout was observed as compared to secretion levels immediately after the exercise bout (*P* = 0.073). MMP-9 secretion upon LPS and IFN-*γ* stimulation, on the other hand, rapidly and immediately increased following acute physical activity (*P* = 0.016) and remained highly inducible after the 2-hour resting phase in patients and controls (*P* = 0.017, Figure 5). No effect of acute exercise on IL-1*β*, IL-6, IFN-*α*, and caspase-1 secretion following LPS and IFN-*γ* stimulation could be demonstrated. Similarly, no effect of acute exercise was found on the secretion of IL-1*β*, IL-6, IL-12p70, TNF-*α*, IFN-*α*, MMP-9, and caspase-1 following IQ stimulation.

## 4. Discussion

In this study, we have demonstrated a rapid and immediate increase in absolute leukocyte number, including lymphocytes and granulocytes, upon one exercise bout in MS patients and healthy controls, which is in line with previous findings by others [1]. Interestingly, the number of lymphocytes and granulocytes remained high after a 2-hour recovery period, although the total number of leukocytes normalized again. Furthermore, in MS patients also the monocyte count increased immediately after the exercise bout and recovered to steady-state values after two hours of rest, while healthy controls failed to show a significant response in monocyte numbers to one exercise bout. Interestingly, the higher the patient's EDSS score, the less pronounced the total leukocyte and monocyte response, which is possibly a consequence of a less intensive exercise due to greater immobility in these patients [32, 33]. Furthermore, also the absolute number of leukocyte subsets with well-described immunoregulatory functions, namely, Treg and cDC and pDC, was immediately elevated upon one exercise bout in MS patients and healthy controls, in agreement with other studies [34–36].

Since we and others previously reported that DC may play an important role in the immunopathogenesis of MS [13, 14, 23], we aimed here to better understand the mechanisms that may underlie DC accumulation in the peripheral blood following acute physical activity. Interestingly, increased levels of Flt3L, which specifically recruits steady-state cDC and pDC towards the circulation [37–39], were found in patients and controls upon one exercise bout. Flt3L-mobilized blood DC were previously shown to migrate towards CCR5 ligands [40]. In accordance with this, increased numbers of cDC and pDC expressing CCR5 upon one exercise bout in patients and controls were found in this study. In addition, increased numbers of cDC and pDC expressing the cell adhesion molecule CD62L were found upon acute exercise in patients and controls. Furthermore, the MMP-9 serum level increased immediately upon acute exercise in controls, as demonstrated by others [41, 42], while patients only show an increase after two hours of recovery. The increased secretion of MMP-9 immediately after acute exercise in patients and controls upon LPS and IFN-*γ* stimulation suggests that MMP-9 found in serum is, at least in part, produced by stimulated DC [43]. Others previously demonstrated exercise-induced leukocyte mobilization from the marginal pool towards the circulation [44]. Here, we suggest that circulating Flt3L might specifically mobilize steady-state CCR5-expressing cDC and pDC from the marginal pool upon acute exercise, as indicated by migration of Flt3L-mobilized blood DC towards CCR5 ligands [40]. Given its function in transendothelial migration [45], also CD62L expression on cDC and pDC may contribute to their mobilization towards the circulation driven by MMP-9-dependent remodelling of the vascular endothelium comprising the marginal pool [46]. Indeed, MMP-9 was previously shown to be involved in stem cell mobilization from the bone marrow by remodelling of the matrix and basement membranes [47], as well as in vascular remodelling in murine models [48]. Overall, we propose that rapid DC cell recruitment from the marginal pool upon physical exercise involves cell-surface expression of the migratory molecules, CCR5 and CD62L, and is mediated by migration-promoting mediators, such as Flt3L and MMP-9.

Furthermore, our results suggest that cDC and pDC from MS patients and healthy controls are less responsive to TLR stimulation after one exercise bout. Indeed, upregulation of CD86 expression on cDC following TLR stimulation was less pronounced in both patients and controls after the 2-hour recovery phase as compared to steady-state values. In addition, upregulation of HLA-DR expression on pDC following TLR stimulation was less pronounced in both patients and controls immediately after one exercise bout. Similarly, Lancaster et al. showed lower upregulation of CD80, CD86, and MHC class II expression on monocytes from healthy volunteers following TLR activation in samples obtained immediately after and following 2 hours of recovery from a 1.5-hour strenuous exercise in comparison with samples obtained at rest [5]. On the other hand, we observed increased secretion of IL-12p70 and TNF-*α* immediately after acute exercise in patients and controls upon LPS and IFN-*γ* stimulation. Since TNF-*α* and IL-12p70 can be secreted by cDC [23, 49], we propose here that the observed increase in the number of cDC, at least in part, contributes to the increased secretion of inflammatory mediators upon LPS and IFN-*γ* stimulation after acute exercise, rather than increased secretion per individual cDC, which is in line with the above-mentioned reduced upregulation of costimulatory markers. This hypothesis is supported by findings of others demonstrating that an absolute increase in monocyte number was responsible for the increased TLR-mediated proinflammatory cytokine production after physical exercise. In fact, these monocytes produced less cytokine per cell [4, 5, 50]. Future studies are, however, needed to support TLR-mediated secretion of inflammatory cytokines using intracellular flow cytometry in order to characterize the identity of the cytokine-producing cell types as well as the amount of cytokine produced per cell after one exercise bout.

In conclusion, our results indicate accumulation of DC in the peripheral blood after one exercise bout which, at least in part, is mediated by a Flt3L- and MMP-9-mediated process and involves cell-surface expression of the migratory molecules, CCR5 and CD62L. Further elucidation of the mechanisms involved may shed light on current knowledge regarding the pathologic role of DC in various inflammatory diseases. Moreover, our results demonstrate a reduced TLR responsiveness of cDC and pDC after acute physical activity, indicating that DC are less prone to drive inflammatory processes following exercise [1, 2]. Together with the observed increase of Treg numbers upon one exercise bout, our findings may present a negative feedback mechanism for the immune system's ability to induce tissue damage and inflammation following exercise. Ultimately, this may provide a tool to modulate the underlying disease pathogenesis of MS contributing to the long-term health benefits of regular exercise [2].

## Supplementary Material

Supplementary Table 1. Study subjects and disease characteristics.
Supplementary Figure 1. Patients with a high EDSS score demonstrate a less pronounced increase in leukocyte and monocyte numbers following one exercise bout.

## Figures and Tables

**Table 1:** Clinical details of the study population.

	Controls	MS	*P* values
Gender (M/F)	4/5	10/12	0.960
Age ± SEM	46 ± 3	46 ± 2	0.960
BMI ± SEM	25 ± 1	24 ± 1	0.424
EDSS ± SEM	NA	3 ± 0.2	NA
Type MS (CP/RR)	NA	9/12^*∗*^	NA
Medication (untreated/1st-line treatment/2nd-line treatment)	NA	4/12/6	NA

Patients were defined as untreated when a wash-out period of at least 3 months was respected before recruitment in the study. 1st-line treatment: IFN-*β* (Avonex, Betaferon, and Rebif) and glatiramer acetate (Copaxone); 2nd-line treatment: alemtuzumab (Campath), natalizumab (Tysabri), and fingolimod (Gilenya). Results are shown as mean ± SEM. ^*∗*^Missing data from one patient.

Abbreviations used are as follows: MS: multiple sclerosis; M: male; F: female; BMI: body mass index; EDSS: expanded disability status scale; CP: chronically progressive MS; RR: relapsing-remitting MS; SEM: standard error of the mean; NA: not applicable.
