# Supplementary material for: Rapid Exercise-Induced Mobilization of Dendritic Cells Is Potentially Mediated by a Flt3L- and MMP-9-Dependent Process in Multiple Sclerosis
Source: Mediators Inflamm. 2015 Oct 29;2015:158956. doi: 10.1155/2015/158956 (PMC4641936; doi:10.1155/2015/158956)
Supplement: Supplementary file 1 — Supplementary Table 1. Study subjects and disease characteristics. Supplementary Figure 1. Patients with a high EDSS score demonstrate a less pronounced increase in leukocyte and monocyte numbers following one exercise bout. [file 158956.f1.doc]

**Supplementary Table 1. Study subjects and disease characteristics**

| **MS patients (n = 22)** | | | | | | | **Healthy controls (n = 9)** | | | |
| --- | --- | --- | --- | --- | --- | --- | --- | --- | --- | --- |
| **ID** | **Type MS** | **EDSS** | **MS medication** | **Age** | **Gender** | **BMI** | **ID** | **Age** | **Gender** | **BMI** |
| **UPN201** | CP | 3 | Betaferon®, Tysabri® | 56 | M | 23 | **UPN001** | 49 | F | 26 |
| **UPN202** | CP | 6 | None | 49 | F | 19 | **UPN002** | 49 | M | 23 |
| **UPN203** | CP | 4 | Avonex® | 40 | M | 33 | **UPN003** | 31 | M | 24 |
| **UPN205** | RR | 2 | Avonex® | 50 | M | 25 | **UPN004** | 53 | M | 26 |
| **UPN211** | RR | 1.5 | Tysabri® | 38 | F | 24 | **UPN005** | 44 | F | 27 |
| **UPN218** | CP | 4 | None | 51 | F | 20 | **UPN006** | 47 | M | 30 |
| **UPN219** | CP | 2.5 | Avonex® | 65 | M | 24 | **UPN007** | 34 | F | 23 |
| **UPN224** | RR | 1.5 | Tysabri® | 27 | M | 25 | **UPN008** | 63 | F | 23 |
| **UPN225** | RR | 2.5 | Rebif® | 52 | F | 17 | **UPN009** | 48 | F | 24 |
| **UPN226** | RR | 1 | Rebif®, Avonex®, Copaxone® | 39 | F | 38 |  |  |  |  |
| **UPN227** | RR | 3 | Avonex® | 32 | M | 23 |  |  |  |  |
| **UPN232** | CP | 4 | Tysabri®, Avonex® | 45 | F | 19 |  |  |  |  |
| **UPN235** | RR | 2.5 | Rebif®, Copaxone® | 59 | F | 27 |  |  |  |  |
| **UPN236** | RR | 2.5 | Avonex® | 41 | F | 21 |  |  |  |  |
| **UPN237** | CP | 4 | Campath® | 35 | M | 22 |  |  |  |  |
| **UPN238** | CP | 4 | Copaxone® | 51 | F | 32 |  |  |  |  |
| **UPN240** | RR | 3 | Rebif®, Campath® | 41 | F | 20 |  |  |  |  |
| **UPN241** | RR | 3 | Betaferon® | 56 | M | 28 |  |  |  |  |
| **UPN244** | RR | 3.5 | None | 48 | M | 24 |  |  |  |  |
| **UPN246** | / | / | Rebif® | 48 | F | 24 |  |  |  |  |
| **UPN249** | RR | 2 | None | 51 | F | 19 |  |  |  |  |
| **UPN250** | CP | 4 | Avonex® | 49 | M | 21 |  |  |  |  |
|  | CP/RR:  9/12 | Mean: 3  SEM: 0.2 |  | Mean: 46  SEM: 2 | M/F:  10/12 | Mean: 24  SEM: 1 |  | Mean: 46  SEM: 3 | M/F:  4/5 | Mean: 25  SEM: 1 |

Patients were defined as untreated when a wash-out period of at least 3 months was respected before recruitment in the study. 1^st^-line treatment: IFN-β (Avonex®, Betaferon®, Rebif®) and glatiramer acetate (Copaxone®); 2^nd^-line treatment: alemtuzumab (Campath®), natalizumab (Tysabri®) and fingolimod (Gilenya®).

Abbreviations used: MS, multiple sclerosis; RR, relapsing-remitting MS; CP, chronically-progressive MS; UPN, unique patient number; EDSS, expanded disability status scale; BMI, body mass index; M, male; F, female; SEM, standard error of the mean.

**Supplementary Figure 1. A high EDSS score results in a less pronounced increase in leukocyte and monocyte numbers following one acute exercise**

**
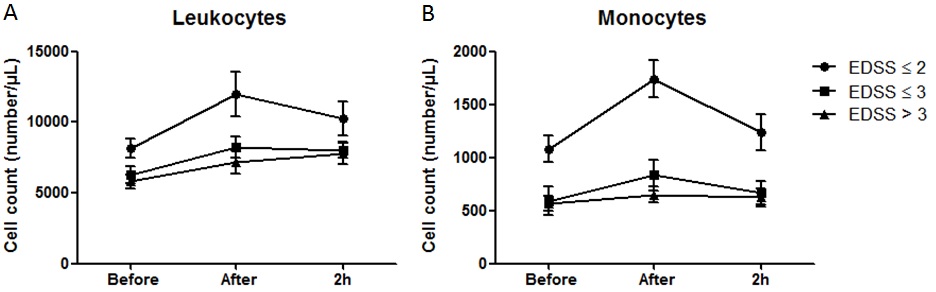
**

Enumeration of the absolute number of immune cells was done by means of a double platform method using an automated cell counter and flow cytometry. EDSS score significantly affects the response of leukocyte and monocyte numbers to acute exercise in MS patients. The higher the patient’s EDSS score, the less pronounced the increase in leukocyte and monocyte numbers following one acute exercise. Results are shown as mean concentration ± SEM from MS patients grouped according to their EDSS score, *i.e.* EDSS ≤ 2 (n = 5), EDSS ≤ 3 (n = 8) and EDSS > 3 (n = 8).

Abbreviations used: EDSS, expanded disability status scale; Before, measurement before the exercise test; After, measurement immediately after the test; 2h, measurement 2 hours after the test.
